# Full characterization of the three pathways of the complement system in patients with systemic lupus erythematosus

**DOI:** 10.3389/fimmu.2023.1167055

**Published:** 2023-04-21

**Authors:** María García-González, Fuensanta Gómez-Bernal, Juan C. Quevedo-Abeledo, Yolanda Fernández-Cladera, Agustín F. González-Rivero, Antonia de Vera-González, Iñigo de la Rua-Figueroa, Raquel López-Mejias, Federico Díaz-González, Miguel Á. González-Gay, Iván Ferraz-Amaro

**Affiliations:** ^1^ Division of Rheumatology, Hospital Universitario de Canarias, Tenerife, Spain; ^2^ Division of Central Laboratory, Hospital Universitario de Canarias, Tenerife, Spain; ^3^ Division of Rheumatology, Hospital Doctor Negrín, Las Palmas de Gran Canaria, Spain; ^4^ Epidemiology, Genetics and Atherosclerosis Research Group on Systemic Inflammatory Diseases, Hospital Universitario Marqués de Valdecilla, Instituto de Investigación sanitaria Marqués de Valdecilla (IDIVAL), Santander, Spain; ^5^ Department of Internal Medicine. University of La Laguna (ULL), Tenerife, Spain; ^6^ Division of Rheumatology, Instituto de Investigación Sanitaria Fundación Jiménez Díaz (IIS-FJD), Madrid, Spain; ^7^ University of Cantabria, Instituto de Investigación sanitaria Marqués de Valdecilla (IDIVAL), Santander, Spain; ^8^ Cardiovascular Pathophysiology and Genomics Research Unit, School of Physiology, Faculty of Health Sciences, University of the Witwatersrand, Johannesburg, South Africa

**Keywords:** systemic lupus erythematosus, complement system, complement pathways, disease activity, disease damage, disease profiles

## Abstract

**Background:**

To date a complete characterization of the components of the complement (C) pathways (CLassical, LEctin and ALternative) in patients with systemic lupus erythematosus (SLE) has not been performed. We aimed to assess the function of these three C cascades through functional assays and the measurement of individual C proteins. We then studied how they relate to clinical characteristics.

**Methods:**

New generation functional assays of the three pathways of the C system were assessed in 284 patients with SLE. Linear regression analysis was performed to study the relationship between the activity, severity, and damage of the disease and C system.

**Results:**

Lower values of the functional tests AL and LE were more frequent than those of the CL pathway. Clinical activity was not related to inferior values of C routes functional assays. The presence of increased DNA binding was negatively linked to all three C pathways and products, except for C1-inh and C3a which were positively related. Disease damage revealed a consistent positive, rather than a negative, relationship with pathways and C elements. Anti-ribosomes and anti-nucleosomes were the autoantibodies that showed a greater relationship with C activation, mainly due to the LE and CL pathways. Regarding antiphospholipid antibodies, the most related with C activation were IgG anti-β2GP, predominantly involving the AL pathway.

**Conclusion:**

Not only the CL route, but also the AL and LE are related to SLE features. C expression patterns are linked to disease profiles. While accrual damage was associated with higher functional tests of C pathways, anti-DNA, anti-ribosomes and anti-nucleosomes antibodies, were the ones that showed a higher relationship with C activation, mainly due to the LE and CL pathways.

## Introduction

The complement (C) system is a key component of innate immunity and a “complement” (hence its name) for antibody-triggered responses. It consists of almost 60 plasma and membrane proteins that form three distinct but overlapping pathways of activation, as well as a common terminal lytic cascade and a network of regulators and receptors (1). There are three major pathways of complement activation: the classical (CL), the alternative (AL) and the lectin (LE), pathways. The CL pathway is initiated by antibody-dependent as well as antibody-independent mechanisms leading to the formation of C1 proteolytic complex; the AL pathway is characterized by plasma tonic low-level C3 activation by hydrolysis in a process termed “tick-over”; and the LE pathway is initiated by target recognition through mannose-binding lectin and ficolins that are proteins components involved in host responses against foreign organisms such as bacteria and viruses. The main functions of C include, among others, recognition and clearance of foreign pathogens and antigens, phagocytosis of opsonized targets, and promotion and modulation of humoral immune responses (1).

Systemic lupus erythematosus (SLE) is a chronic, occasionally life-threatening, multisystem immune-mediated disorder. Patients can present with a wide range of symptoms, signs, and laboratory findings and have a variable prognosis depending on the severity of the disease and the type of organ affected. Hypocomplementemia is a typical laboratory finding in patients with SLE, reflecting in most cases activation of the C system by immune complexes. Accelerated consumption exceeds synthesis, being the main cause of the hypocomplementemia (2). In this setting, low C values tend to correlate with more severe SLE, especially renal disease, and with antibodies to double-stranded DNA, while return to normal levels with treatment is a good prognostic sign (3). However, routine C assessment in the clinical setting is generally restricted to the measurement of inactive C3 and C4 zymogens, without considering activated or regulatory molecules. Since many other factors besides consumption can affect the serum levels of C proteins, the only evaluation of the C3 and C4 values cannot be considered a surrogate marker of C activity in SLE (4).

Although it is becoming more commercially available, neither functional C assays nor individual plasma C components other than C3 and C4 are routinely used by most experienced lupus clinicians. In this regard, the medical literature lacks studies in which a complete characterization of the C system in patients with SLE has been performed. In the present work, we have evaluated the three C-system pathways through next-generation functional assays in a well-characterized series of SLE patients with a diverse set of organic manifestations. In addition, we have measured C components belonging to all three C pathways, including proteolytically derived fragments and serum regulators of the C system. Our aim was to identify how the functional levels of the three C pathways, and specific C elements of these pathways relate to damage, severity, and activity of the disease, as well as to individual characteristics of the disease.

## Material and methods

### Study participants

This was a cross-sectional study that included 284 patients with SLE. All patients with SLE were 18 years or older, had a clinical diagnosis of SLE, and met ≥ 4 American College of Rheumatology (ACR) classification criteria for SLE (5). Patients were recruited since 2016 to 2021. They had been diagnosed by rheumatologists and were regularly followed up in rheumatology outpatient clinics. Patients were excluded if they had a history of cancer, chronic liver and/or renal failure, evidence of acute and/or chronic active infection, and/or any other chronic autoimmune disease other than a condition such as antiphospholipid and/or Sjögren’s syndrome associated with SLE. Research was carried out in accordance with the Declaration of Helsinki. The study protocol was approved by the Institutional Ethics Committees of the Hospital Universitario de Canarias and the Hospital Universitario Doctor Negrín (both in Spain), and all subjects provided informed written consent (Approval Number 2015_84).

### Data collection

Patients included in the study completed a medication use questionnaire and underwent a physical examination. Medical records were reviewed to verify specific diagnoses and medications. SLE disease activity and damage were assessed using the Systemic Lupus Erythematosus Disease Activity Index -2000 (SLEDAI-2K) (6) and the Systemic Lupus International Collaborating Clinics/American College of Rheumatology (SLICC/ACR) Damage Index -SDI- (7), respectively. For the purpose of the present study, the SLEDAI-2k index was divided into none (0 points), mild (1-5 points), moderate (6-10 points), high (11–19) points, and very high activity (≥20) points as previously described (8). The severity of the disease was measured using the Katz index (9). Fasting serum samples were collected and frozen at -80°C until analysis of C system.

### Laboratory assessments

The SVAR functional C assays under the Wieslab^®^ brand (Sweden) were used to assess CL, AL and LE pathways activity. These tests combine principles of the hemolytic assay for C function with the use of labelled antibodies specific for the neoantigen produced as the result of C activation. The amount of neoantigen generated is proportional to the functional activity of C pathways. Microtiter strip wells are coated with CL, LE, or AL pathway-specific activators. The patient’s serum is diluted in a diluent containing a specific blocker to ensure that only the studied pathway is activated. During the incubation of the diluted patient serum in the wells, the specific coating activates C. The wells are then washed, and C5b-9 is detected with an alkaline phosphatase labeled specific antibody against the neoantigen expressed during membrane attack complex (MAC) formation. After an additional washing step, detection of specific antibodies is obtained by incubation with alkaline phosphatase substrate solution. The amount of C activation correlates with the intensity of the color and is measured in terms of absorbance (optical density). The amount of formed MAC (neo-epitope) reflects the activity of the C cascade. The result is expressed semi-quantitatively using the optical density ratio between a positive control and the sample. Wieslab^®^ has validated these functional assays by studying their correlation and concordance with the classical CH50 and AH50 hemolytic tests (https://www.svarlifescience.com/). C2, C3, C3a, C4 and C1q were analyzed by turbidimetry (Roche), C1-inhibitor was analyzed through nephelometry (Siemens) whereas factor D and factor H were assessed by enzyme linked immunosorbent assay (ELISA Duoset, R&D). Both intra and inter-coefficients of variability were < 10% for these assays.

### Statistical analysis

Demographic and clinical characteristics were described as mean ± standard deviation (SD) or percentages for categorical variables. For non-normally distributed continuous variables, data were expressed as median and interquartile range (IQR). The relationship of SLE features with circulating C system molecules and pathways was assessed through linear regression analysis. All the analyses used a 5% two-sided significance level and were performed using Stata software, version 17/SE (StataCorp, College Station, TX, USA). P-values <0.05 were considered statistically significant.

## Results

### Demographic and disease-related data of patients with systemic lupus erythematosus

Demographic and disease-related characteristics of patients with SLE are shown in **Table 1**. Most of them were women (92%) and the mean age ± SD was 50 ± 12 years. The age of diagnosis was 34 ± 13 years, and the duration of the disease was 16 ± 10 years. At the time of recruitment, 67% of the patients were positive for anti-DNA and 69% for ENA, with anti-SSA being the most frequently found antibody (35%). Sixteen percent of patients met the definition of associated antiphospholipid syndrome, and 32% had at least one positive antiphospholipid antibody. The majority of patients with SLE were in the categories of no activity (40%) or mild-moderate activity (55%) as shown by the SLEDAI-2K score. SDI and Katz indexes were 1 (IQR 0-2) and 2 (IQR 1-4), respectively. Sixty-eight percent of the patients had a SDI score equal to or higher than 1. Regarding treatments at the time of assessment, half of the patients (50%) were taking glucocorticoids and the median equivalent daily dose of prednisone was 5 mg/day (IQR 5-7.5 mg). Sixty-nine percent of the patients were taking hydroxychloroquine when the study was performed. Other less used drugs were methotrexate (11%) and azathioprine (15%). **Table 1** shows additional information on the data related to SLE.

**Table 1:** Demographic and disease-related data of patients and controls.

	SLE (n=284)	
Age, years	50 ± 12	
Women, n (%)	261 (92)	
Smoking, n (%)	69 (24)	
Diabetes, n (%)	16 (6)	
Hypertension, n (%)	111 (39)	
Obesity, n (%)	85 (30)	
SLE related data		
Age at diagnosis, years	34 ± 13	
Disease duration, years	16 ± 10	
SLE classification criteria*, n (%)	150 (88)	
Antiphospholipid syndrome, n (%)	43 (16)	
Auto-antibody profile		
Anti DNA positive, n (%)	151 (67)	
ENA positive, n (%)	164 (69)	
Anti-Sm	24 (10)	
Anti-ribosome	13 (9)	
Anti-nucleosome	32 (22)	
Anti-histone	22 (15)	
Anti-RNP	64 (28)	
Anti-SSA/Ro	55 (35)	
Anti-SSB/La	36 (21)	
Any antiphospholipid antibody, n (%)	61 (32)	
ACA IgM	22 (11)	
ACA IgG	39 (20)	
Anti beta2 glycoprotein IgM	19 (10)	
Anti beta2 glycoprotein IgG	28 (15)	
Disease scores		
Median SLEDAI-2K	2 (0-4)	
SLEDAI-2K categories		
No activity, n (%)	109 (40)	
Mild, n (%)	107 (40)	
Moderate, n (%)	41 (15)	
High or Very High, n (%)	14 (5)	
Median SDI	1 (0-2)	
SDI ≥ 1, n (%)	191 (68)	
Katz Index	2 (1-4)	
Katz ≥ 3, n (%)	126 (44)	
Functional complement assays, %		
Classical pathway	91 ± 38	
Alternative pathway	41 (12-79)	
Lectin pathway	10 (1-41)	
Individual complement components		
C1q, mg/dl	34 ± 11	
C2, mg/dl	2.5 ± 1.2	
C4, mg/dl	21 ± 12	
Factor D, ng/ml	2593 ± 1835	
C3, mg/dl	130 ± 40	
C3a, mg/dl	39 ± 10	
C1 inhibitor, mg/dl	32 ± 9	
Factor H, ng/ml x10e-3	388 (281 - 564)	
Immunosuppressants at the time of the visit		
Glucocorticoids, n (%)	140 (50)	
Prednisone equivalent daily dose, mg	5 (5-7.5)	
Antimalarials drugs, n (%)	194 (69)	
Methotrexate, n (%)	31 (11)	
Azathioprine, n (%)	43 (15)	
Mycophenolate mofetil, n (%)	31 (11)	
Belimumab, n (%)	8 (3)	
Rituximab, n (%)	8 (3)	

Data represent mean ± SD or median (interquartile range) when data were not normally distributed.

SLEDAI-2K categories were defined as: 0, no activity; 1-5 mild; 6-10 moderate; >10 high activity.

*Met either ACR1997 or SLICC 2012 classification criteria (ACR: American College of Rheumatology; SLICC: Systemic Lupus International Collaborating Clinics).

ACA, anticardiolipin antibodies; ENA, extractable nuclear antibodies; SDI, SLICC/ACR Damage Index; SLE, systemic lupus erythematosus;. SLEDAI-2K, SLE Disease Activity Index.

Functional C assays of the CL, AL and LE pathways were 91 ± 38%, 41 (IQR 12-79) % and 10 (IQR 1-41) %, respectively. Single C components, C1q, C2, C3, C3a, C1-inhibitor (C1-inh), and factor D and H serum values are shown in **Table 1**.

A graphical representation of the frequency distribution of the three C pathways functional assays is additionally shown in **Supplementary Figure 1**. In this figure, X axes represents the value of the functional assay, and Y axes is the number of patients with a given value. As it can be observed, while the CL pathway functional test was normally distributed, both AL and LE were skewed to the left toward lower values. Correlations between C routes functional assays and individual components are shown in **Supplementary Table 1**, and **Supplementary Figures 2** (scatterplots of C functional assays and C3 and C3a) and 3 (chord diagrams). C functional assays and molecules were positively and highly correlated with each other, except for factors D and H, which showed a non-significant relationship with C routes and products (**Supplementary Table 1**). A chord diagram showing a many-to-many relationship between C elements, and routes, as curved arcs within a circle is illustrated in **Supplementary Figure 3**. Thickness of the arcs are proportional to the significance of the flow. As it can be seen, flows or connections between nodes did not show a specific pattern of C activation, since all C elements generally correlated to each other in a similar manner (**Supplementary Figure 3**).

### Complement pathway activity and relationship of individual proteins to indices of activity, damage, and disease severity in SLE

The relationship of the SLEDAI-2K, SDI, and Katz indices to the three C pathway functional assays and C products is shown in **Table 2**. In these analyses, the scores are the independent variable and are considered both continuous and categorized (SLEDAI-2K inactive, mild, and moderate to very high; SDI equal to or greater than 1; and Katz equal to or greater than 3). With respect to SLEDAI-2K, in general, this score was related to lower values of the C pathway tests and products. In particular, C2, C3 and C1q and AL route were the ones that revealed a higher negative relationship with both continuous and categorized crude SLEDAI-2K. In addition, SLEDAI-2K was also associated with lower C3a values in the comparison between the mild and inactive categories. In contrast, C1-inh and factors D and H did not reveal associations with crude SLEDAI-2K.

**Table 2:** Complement pathways activity and individual components relation to activity, damage and severity indices.

Beta coefficients (95% confidence interval), p			
	CLASSICAL PATHWAY		LECTIN PATHWAY
	Classical, %	C1q, mg/dl	Lectin, %
SLEDAI-2K	-0.6 (-2-0.5), 0.26	**-0.3 (-0.6- -0.003), 0.048**	0.1 (-1-1), 0.85
Inactive	–	–	–
Mild	**-15 (-25- -5), 0.004**	**-3 (-6- -0.1), 0.042**	**-13 (-25- -2), 0.023**
Moderate to very high	**-13 (-26- -0.6), 0.039**	-3 (-7-0.2), 0.062	-0.8 (-15-13), 0.92
Clinical SLEDAI-2K	0.8 (-2-3), 0.58	0.5 (-0.2-1), 0.16	2 (-1-5), 0.20
Inactive	–	–	–
Mild	-5 (-18-8), 0.49	1 (-2-5), 0.50	4 (-11-19), 0.58
Moderate to very high	10 (-14-35), 0.41	**7 (0.7-13), 0.030**	24 (-3-51), 0.085
SDI	**5 (3-8), <0.001**	**0.88 (0.03-1), 0.042**	**5 (2-8), 0.001**
SDI >=1	**12 (2-22), 0.016**	**3 (0.2-5), 0.038**	6 (-5-17), 0.27
Katz	2 (-1-4), 0.14	-0.2 (-0.8-0.4), 0.55	2 (-0.5-5), 0.11
Katz >=3	4 (-5-13), 0.38	-2 (-4-0.8), 0.18	4 (-6-14), 0.44
COMMON ELEMENTS OF THE CLASSICAL AND LECTIN PATHWAYS			
	C2, mg/dl	C4, mg/dl	C1 inh, mg/dl
SLEDAI-2K	**-0.05 (-0.08- -0.02), 0.004**	-0.3 (-0.7-0.01), 0.058	0.2 (-0.08-0.5), 0.17
Inactive	–	–	–
Mild	**-0.7 (-1.0- -0.4), <0.001**	**-4 (-8- -1), 0.011**	-0.9 (-4-2), 0.47
Moderate to very high	**-0.7 (-1.1- -0.4), <0.001**	**-7 (-11- -3), 0.001**	1 (-2-5), 0.38
Clinical SLEDAI-2K	0.07 (-0.009-0.1), 0.081	0.4 (-0.5-1), 0.41	0.09 (-0.7-0.9), 0.82
Inactive	–	–	–
Mild	0.3 (-0.1-0.7), 0.20	2 (-3-6), 0.42	1 (-2-5), 0.55
Moderate to very high	0.5 (-0.3-1.2), 0.21	4 (-4-11), 0.37	1 (-6-8), 0.74
SDI	**0.09 (0.02-0.2), 0.019**	0.4 (-0.5-1), 0.32	**0.8 (0.1-1), 0.019**
SDI >=1	**0.4 (0.1-0.7), 0.004**	2 (-1-5), 0.25	**3 (0.7-5), 0.011**
Katz	0.0004 (-0.07-0.07), 0.99	0.3 (-0.5-1), 0.45	**0.7 (0.1-1), 0.017**
Katz >=3	-0.1 (-0.4-0.1), 0.31	-1 (-4-2), 0.42	**3 (0.6-5), 0.013**
	ALTERNATIVE PATHWAY		
	Alternative, %	Factor D, ng/ml	
SLEDAI-2K	**-2 (-3- -0.6), 0.002**	-60 (-123-2), 0.057	
Inactive	–	–	
Mild	**-13 (-23- -3), 0.010**	-374 (-931-184), 0.19	
Moderate to very high	**-20 (-33- -8), 0.002**	-552 (-1260-156), 0.13	
Clinical SLEDAI-2K	-0.6 (-3-2), 0.66	-67 (-228-93), 0.41	
Inactive	–	–	
Mild	-10 (-23-3), 0.13	53 (-720-826), 0.89	
ALTERNATIVE PATHWAY			
	Alternative, %	Factor D, ng/ml	
Moderate to very high	1 (-23-25), 0.92	-644 (-2063-776), 0.37	
SDI	2 (-0.1-5), 0.063	108 (-27-242), 0.12	
SDI >=1	**11 (1-20), 0.026**	301 (-204-805), 0.24	
Katz	-0.5 (-2-2), 0.96	65 (-56-186), 0.29	
Katz >=3	-5 (-14-4), 0.29	82 (-413-577), 0.74	
COMMON ELEMENTS OF THE THREE PATHWAYS			
	C3, mg/dl	C3a, mg/dl	Factor H, ng/ml x10e-3
SLEDAI-2K	**-2 (-3- -0.5), 0.006**	0.04 (-0.3-0.3), 0.79	-22 (-44-1), 0.063
Inactive	–	–	–
Mild	**-18 (-29- -7), 0.001**	**-4 (-6- -0.9), 0.010**	-23 (-228-181), 0.82
Moderate to very high	**-27 (-40- -14), <0.001**	-1 (-4-2), 0.57	-236 (-496-23), 0.075
Clinical SLEDAI-2K	2 (-1-4), 0.24	**0.09 (0.2-2), 0.013**	-7 (-66-52), 0.81
Inactive	–	–	–
Mild	8 (-5-22), 0.24	0.3 (-3-4), 0.85	237 (-457-519), 0.10
Moderate to very high	6 (-19-32), 0.62	**11 (4-17), 0.001**	-242 (-761-276), 0.36
SDI	1 (-2-4), 0.43	**1 (0.4-2), 0.001**	5 (-59-72), 0.86
SDI >=1	9 (-1-19), 0.093	**4 (1-6), 0.006**	-93 (-337-151), 0.46
Katz	-0.4 (-3-2), 0.75	0.02 (-0.6-0.6), 0.94	-16 (-75-43), 0.60
Katz >=3	-4 (-14-6), 0.42	1 (-1-4), 0.28	-81 (-32-158), 0.50

Clinical SLEDAI-2K does not account for anti-DNA antibodies or hypocomplementemia items.

SDI: Systemic Lupus International Collaborating Clinics/American College of Rheumatology Damage Index.

SLEDAI2-K: Systemic Lupus Erythematosus Disease Activity Index. SLEDAI categories were defined as: 0, no activity; 1-5 mild; 6-10 moderate; >10 very high.

*inactive SLEDAI-2K category used as reference level.

Statistically significant values are depicted in bold.

Since SLEDAI-2K includes laboratory items (hypocomplementemia and anti-DNA), we additionally calculated the “clinical” SLEDAI-2K in which those two items were not included in the calculation. When the “clinical” - not classical - SLEDAI-2K was tested, most of the significant relationships were lost. This score was only related to higher values of C1q (which belongs to the CL cascade) and higher serum levels of C3a, denoting activation of all three C pathways. (**Table 2**).

Regarding SDI, this score was related to higher values of the functional tests CL, AL and LE, and higher circulating C2, C3a, C1-inh and C1q. No relationship was found between SDI and C3, C4 and factors D and H. The Katz index was not related, in general, with the functional C tests or with the C components. A positive relationship was only found with C1-inh both when considering this score as continuous or binary (**Table 2** and **Supplementary Figure 4**).

### Relationship of SLEDAI-2K and SDI elements to complement pathways and individual proteins

**Figure 1:**
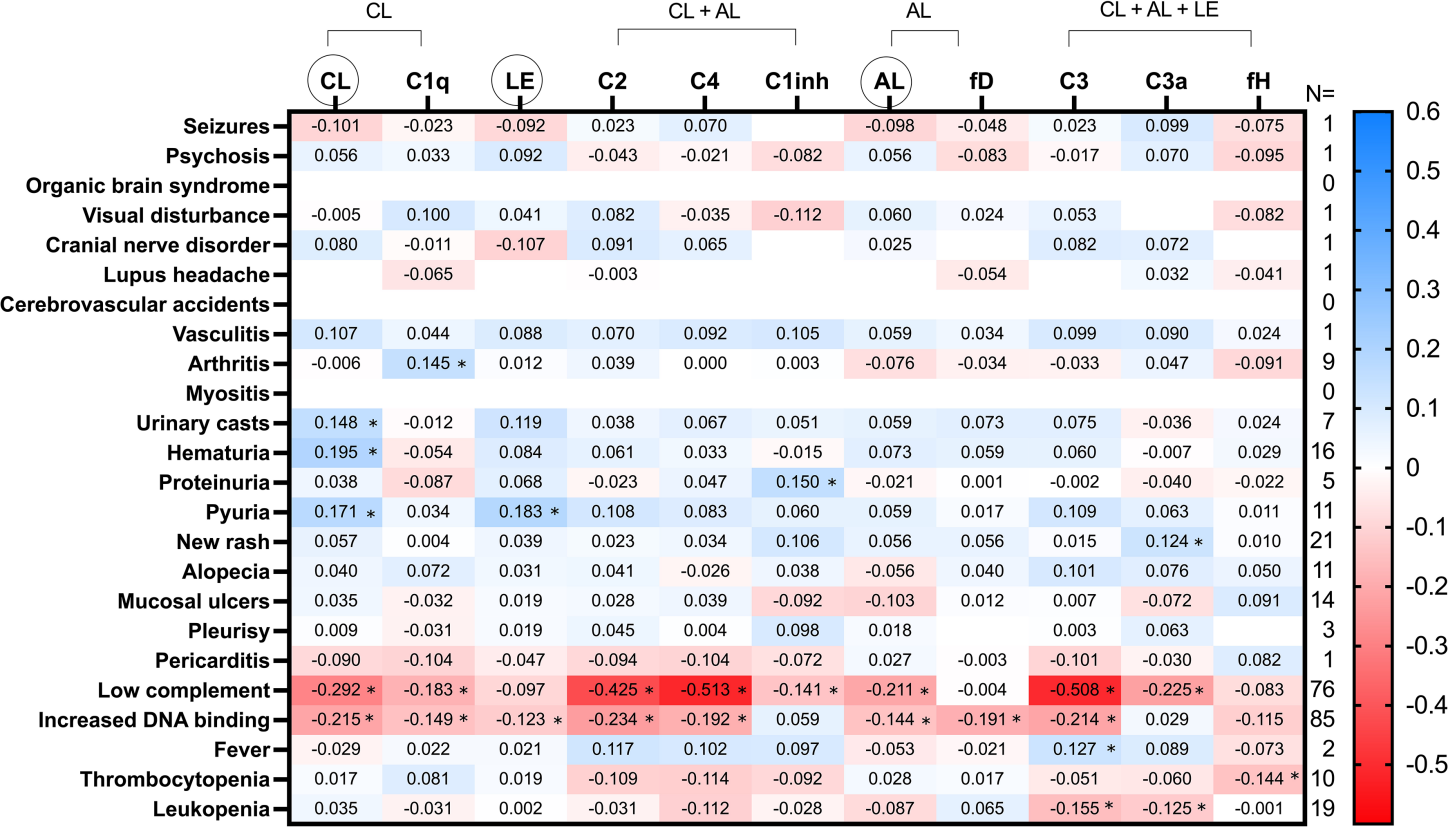
Heatmap of SLEDAI-2K items relation to C pathways activation and serum molecules. Values in the cells represent Spearman’s rho coefficient (* denotes p value < 0.05). Positive and negative correlations are shown in blue and red, respectively. The number of patients who met each SLEDAI-2K Item is shown in the right margin. CL, classical; AL, alternative; LE, lectin; fD, factor D; fH, factor H. CL, LE and AL in circles refer to the functional tests of these cascades.

**Figure 2:**
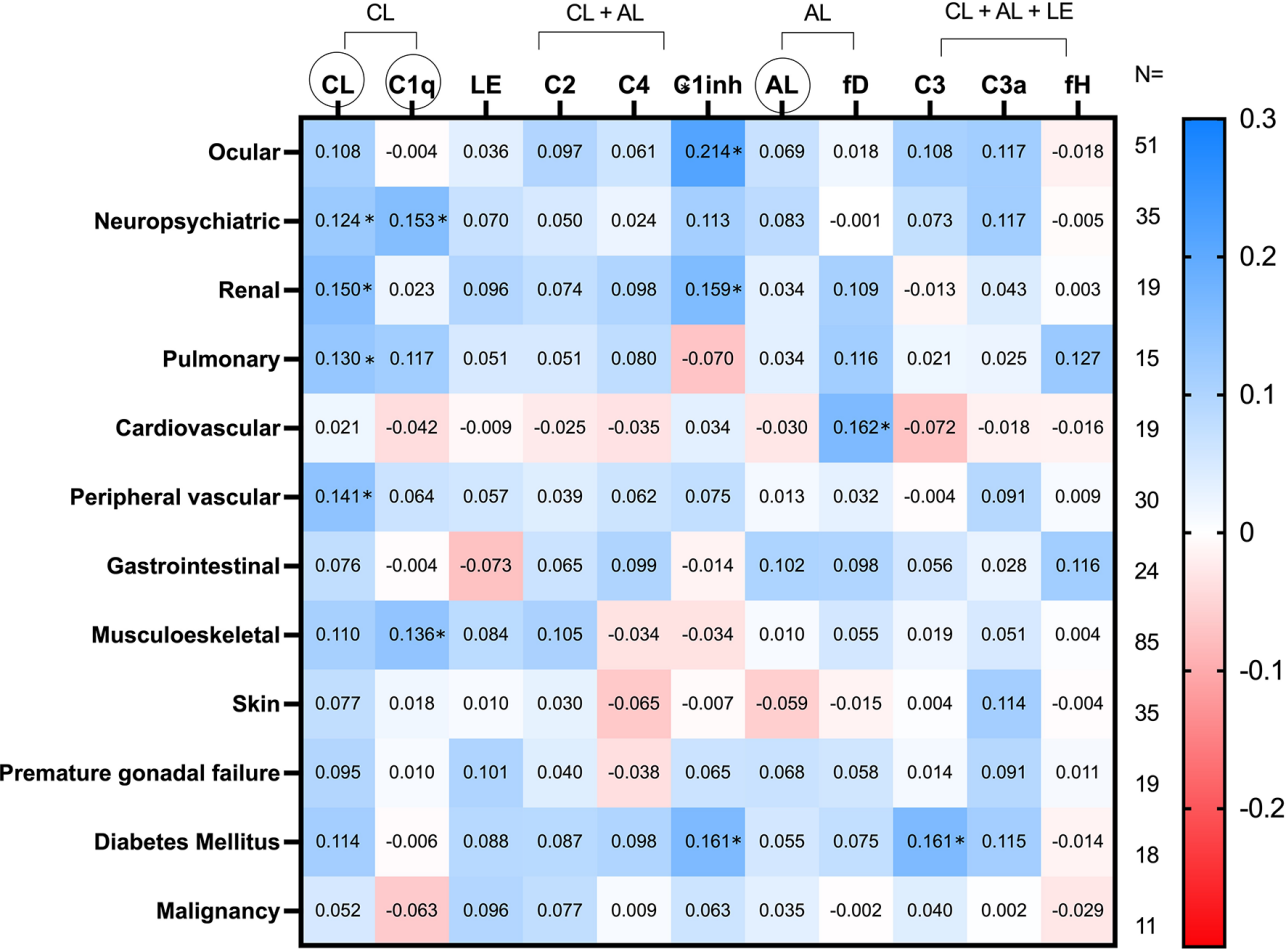
Heatmap of SDI items relationship to C pathways activation and serum molecules. Values in the cells represent Spearman’s rho coefficient (* denotes p value < 0.05). Positive and negative correlations are shown in blue and red, respectively. The number of patients who complied with each item of the SDI is shown in the right margin. CL, classical; AL, alternative; LE, lectin; fD, factor D; fH, factor H. CL, LE and AL in circles refer to the functional tests of these cascades.

A heatmap representation of C pathways and molecules relationship to SLEDAI-2K and SDI items and domains is represented, respectively, in **Figures 1**, **2**. Some C expression patterns can be deduced from these heat maps. Remarkably, the presence of increased DNA binding was negatively related to all three C pathways and products, except for C1-inh and C3a which were positively related. The negative relationship between DNA binding and C was higher for the CL pathway parameters. In addition, low complement, which is defined in the SLEDAI-2K score through the measurement of C3 and C4 in the clinical setting, was associated with lower levels of both C functional assays and products of all three pathways, including the C3a activation product. The CL pathway showed the greatest relationship with the C consumption item.

Items referring to skin, joint, and renal manifestations were associated with higher levels of most of the C parameters. In this sense, the strongest relationship with arthritis was found for C1q in a positive manner. The functional test CL was the one that presented a higher and positive association with most of the renal items. Instead, the hematological features of the disease were related, in general, with lower levels of C values. Regarding regulator molecules, while factor H was mostly related to thrombocytopenia, it was found that the greatest relationship of C1-inh was with the hypocomplementemia item.

With respect to SDI, an overall view of the heatmap of **Figure 2** showed a predominance of blue cells, denoting, therefore, a positive relationship between SDI items and C routes and components. The C parameters that revealed a greater relationship with the CL pathway were the ocular and neuropsychiatric domains, followed by the renal and pulmonary domains. Furthermore, the SDI domain that had the strongest association with the AL cascade was gastrointestinal. Similarly, the C parameters that had the highest correlation with the diabetes and cardiovascular domains were, respectively, C1-inh and factor D. A complete heatmap of all the SDI items is shown in **Supplementary Figure 5**.

### Relationship of autoantibodies to the complement system

**Figure 3:**
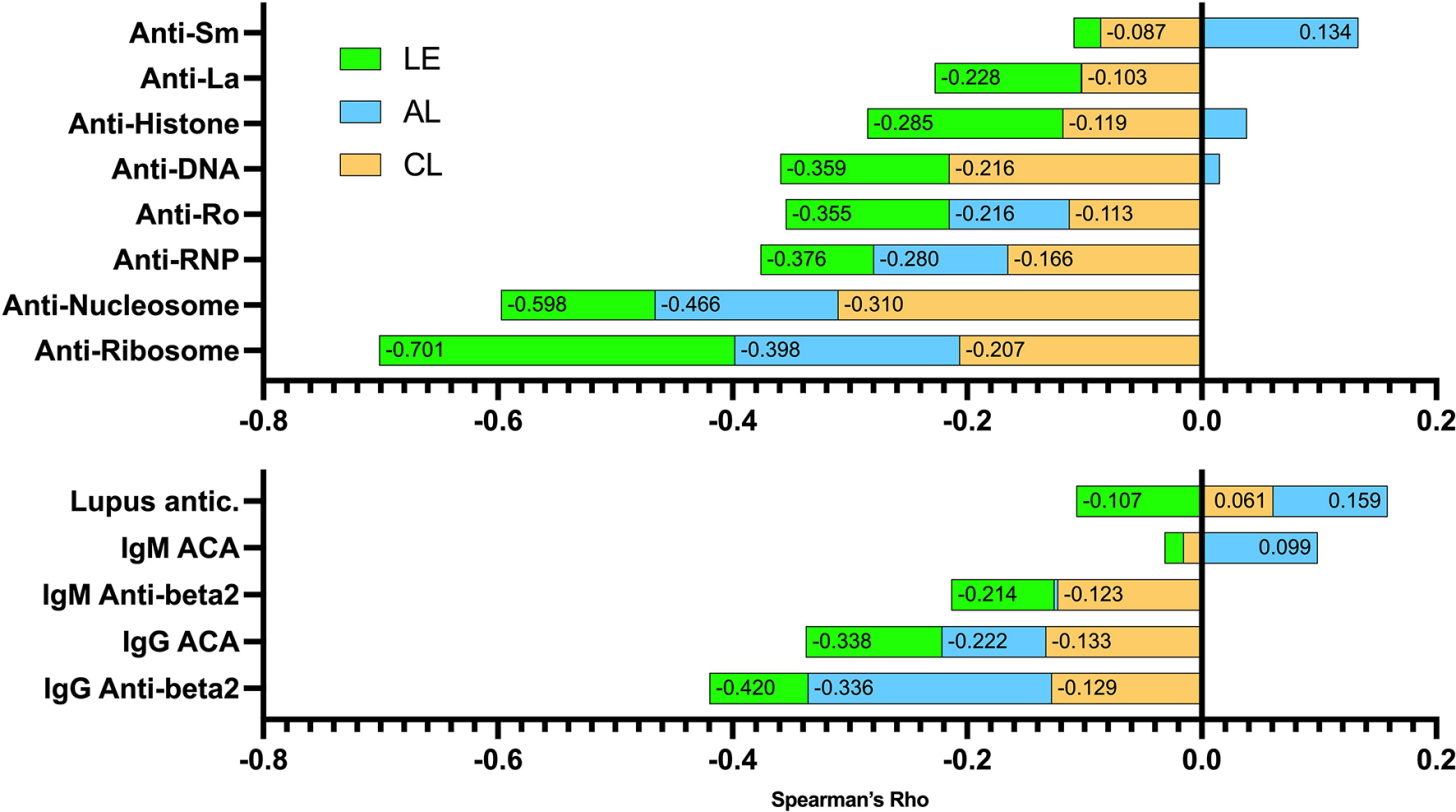
Relationship of autoantibodies to the sum of the Spearman’s rho correlation indices of the three C pathway functional assays. Values in the cells represent Spearman’s rho coefficients. Positive and negative correlations are shown. CL, classical (orange); AL, alternative (blue); LE, lectin (green). ACA, anticardiolipin antibodies; anti-beta2, anti-beta2glycoprotein antibodies; lupus antic.: lupus anticoagulant.

The relationship of ENA, anti-DNA and antiphospholipid autoantibodies to C system is shown in **Supplementary Figure 6** as a heatmap. Moreover, the relationship of these autoantibodies to the sum of C pathways activation is illustrated in **Figure 3**. In this sense, anti-ribosomes and anti-nucleosomes were the autoantibodies that showed a greater relationship with C activation, mainly due to the LE and CL pathways. The anti-La was the ENA least related to the AL pathway. Low relationship with AL was also found with anti-DNA and anti-histone. Regarding antiphospholipid antibodies, the most related with C activation were IgG anti-β2GP, primarily involving the AL pathway.

## Discussion

Our study is the first in the literature in which the three C pathways have been fully characterized in a large set of SLE patients with a wide variety of organic manifestations. Our data support the complexity of the C system in patients with SLE and how it is linked to certain manifestations of the disease. Specifically, while accrual damage is related to superior levels of C individual elements and functional assays, increased DNA binding, and anti-ribosomes and anti-nucleosomes, were the autoantibodies that showed a higher relationship with C activation, mainly due to the LE and CL pathways. According to our findings, the identification of C disturbances may serve as a guide in the therapeutic and prognostic approach of SLE patients.

In our work, according to the frequency distribution of the pathways, the lowest values of the functional tests of the AL and LE pathways were more prevalent than those of the CL pathway. This would imply that the deficit or activation of the AL and LE pathways was more frequent compared to the CL pathway in our population with SLE. This finding of lower values in the LE pathway is consistent with current knowledge of SLE and controls on this pathway. In this regard, deficiencies affecting the LE pathway have been reported to be common, and this may result in lower levels of this LE functional assay in both patients and controls (10). However, to date, functional AL tests have been described to be normally distributed in healthy individuals (11). This was not the case in our SLE patients in which AL curve is skewed to the left towards lower values. In this sense, it is well known that the AL pathway is highly influenced by events that occur in other C pathways. In this regard, AL convertase-C3 amplifies C activation initiated in any pathway, exerting positive feedback to the entire system, and SLE patients more frequently suffer from mutations and autoantibodies that affect C system molecules and regulators. Therefore, we believe that the combination of all this may explain why AL functional test values were skewed to the left reflecting deficit but also activation of this route in SLE patients.

In our analysis of C system in SLE, functional assays were positively related to serum molecules and vice versa. This was also the case for C3a, where a negative correlation would have been expected because this molecule is a proteolytically derived activated element and not an inactive zymogen. We believe that this can be explained by the combination of some evidence: hepatic hyperproduction of complement proteins as acute phase reactants in inflammatory scenarios, spontaneous C3 “tick-over” leading to C3a formation, and secretion of intracellular C3 stores by part of T cells under certain conditions (12). Furthermore, only factors D and H showed non-significant and low relationships with the C pathways or components. One possible explanation could be the presence in SLE of anti-factor H autoantibodies and/or factor H mutations that result in loss of function, as described in other immune-mediated conditions, such as some thrombotic microangiopathies and glomerular diseases (13, 14). Also, factor D, which cleaves factor B, is the only component that can be lost in substantial amounts in the urine. However, in our work an exact amplification pattern or image in SLE could not be described through the representation of chord diagrams. We understand, the high complexity of system C does not allow drawing a clear figure that represents the SLE population.

In our study, the clinical SLEDAI-2K, which does not contain the hypocomplementemia and anti-DNA items, was associated with higher levels of circulating C3a. Despite this, the clinical SLEDAI-2K was not associated with any of the C pathway functional assays or with other serum molecules. This means that, in our work, classical -not clinical- SLEDAI-2K did not capture consumption or alterations in C molecules and pathways. In this regard, it should be remembered that criticism has arisen regarding the SLEDAI-2K score because, for example, only the presence/absence of each item is scored, but its severity cannot be assessed; some elements are over-emphasized while others are under-weighted; and some severe conditions are not captured as activity in this index because there are no items for them (15). According to our results, SLEDAI-2K would not be an optimal tool to capture C disturbances in patients with SLE.

In addition to its key role in host defense, C also promotes inflammation. In this sense, it is known that the activation of the C system leads to the release of “anaphylatoxin” peptides, which are potent mediators of the inflammatory and immune response. These anaphylatoxins bind to their respective receptors on cells to initiate inflammation and vasodilation that in turn activate many cell types (16). Accordingly, C participates in angiogenesis, mobilization of hematopoietic progenitor cells (17) and tissue regeneration (18). Furthermore, C3 activation can occur intracellularly, resulting in the production of autocrine produced proinflammatory cytokines to signal the inflammasome (19). In this regard, C causes the release of mediators, such as interleukin 6, tumor necrosis factor alpha, and soluble vascular endothelial growth factor from multiple cell types, including monocytes and macrophages (20).

Our study evaluated for the first time the relationship of cumulative disease damage with C. Regarding this, disease damage assessed by SDI, which does not contain C-related items, showed in our study a positive relationship with serum C levels and its functional tests. This relationship was found not only with functional assays of all three pathways, but also with various components of all three pathways such as C1q (CL pathway), C2 and C1inh (CL and LE pathways) and C3a (common pathway). Furthermore, when the SDI score was broken down into its different domains and items, the strongest positive relationships were found mainly with the ocular, neuropsychiatric, renal, peripheral vascular, cardiovascular, and diabetes domains. Therefore, we believe that the positive association between the disease damage and the C system is consequence of the inflammation, angiogenesis and tissue repair mechanisms, which has been established due to the accrual damage in different organs.

Regarding the association between SLE and C autoantibodies, some authors have found that anti-Sm, anti-DNA and anti-SSA are related to C3, C4 and/or C3a (13, 21, 22), while others have described a lack of association between anti-RNP autoantibodies and C3 and C4 (23). The relationship of SLE autoantibodies with C functional assays has not been studied until the present work. The fact that these relationships have been evaluated through functional tests, and not using C elements, is of great value. In our study, anti-ribosomes and anti-nucleosomes were the antibodies that had the greatest relationship with the functional tests of C.

It is known that the complement system and coagulation are related. In fact, there is great interference between coagulation and C, so activation of one system can amplify the other (24). For example, higher levels of C5b-9 are reported in patients with antiphospholipid syndrome, many patients with antiphospholipid syndrome have hypocomplementemia and/or elevated levels of C activation products Bb and C3a (25, 26). In addition, C activation has also been reported in patients with isolated antiphospholipid antibodies or primary antiphospholipid syndrome unrelated to SLE (25). In this sense, it is striking that in our study the antiphospholipid antibody most related to C activation was the anti-β2GP IgG subtype. This is consistent with the fact that this autoantibody is considered etiopathogenic, and the one with a higher relation to clinical manifestations in antiphospholipid syndrome (27).

Dysregulation of the C system is now known to play an important role in various diseases, from autoimmune conditions and sepsis to neurodegenerative disorders and graft rejection. For this reason, an increasing number of pharmaceutical companies are focusing on developing drugs that regulate the C system at different levels and the field of research is growing every year in areas where C is suspected to be a pathological problem, which raises the question if treatment options within system C are needed. We believe that a better understanding of the role of C in SLE, as shown in our study, could pave the way for the development of therapies related to the C system in patients with SLE. However, it should be noted that our study is mainly exploratory. Correlations of disease manifestations with C pathways and molecules are shown descriptively. The fact that the correlations were sometimes not significant should not be interpreted as a limitation since several manifestations of the disease are rare and infrequent. Our representation through heatmaps aims to describe patterns of activation of the C system for certain manifestations of the disease.

In our study we used novel assays that analyzed C activity based on enzyme immunoassay technology and not through the traditional haemolytic based methods. These new assays have been validated and correlate well with the classical haemolytic ones. Besides, it is well known that the ELISA format offers superior ease of handling, increased objectivity in interpretation, faster turnaround time and increased stability and reagent quality as compared to haemolytic assays. Moreover, the ELISA is suitable for automation which further adds to the ease of use. Reports that use this technology in several diseases and conditions have become frequent in the literature. For example, these assays have been used to assess C function in vasculitis (28, 29), in the study of C deficiencies (30), response to treatments in conditions like retinopathies and coronavirus infection (30, 31) and other diseases (32).

We acknowledge the limitation that our study has a cross-sectional design and therefore causality cannot be inferred. Besides, the prospective implications that the C abnormalities found in our study may have in disease expression warrant further studies in the future. Genetic deficiencies of many C components are strongly associated with the development of SLE and influence the disease. In this regard, we also acknowledge that we have not performed a genetic evaluation of the C system or studied the presence of antibodies against C particles. A potential limitation of our study could be that we did not test C in healthy controls and focused specifically on patients with SLE, as these patients are known to have C abnormalities.

In conclusion, our study demonstrates the complexity of the C system in patients with SLE. All three pathways, not just the CL pathway, appear to be disrupted in SLE patients. Alterations of the C system may contribute to the expression of the disease in terms of serological and clinical manifestations, activity, and damage.

## Data availability statement

The original contributions presented in the study are included in the article/**Supplementary Material**. Further inquiries can be directed to the corresponding authors.

## Ethics statement

The studies involving human participants were reviewed and approved by Institutional Review Committee at Hospital Universitario de Canarias and Hospital Universitario Doctor Negrín. The patients/participants provided their written informed consent to participate in this study.

## Author contributions

Conceptualization: IF-A, MAG-G, MG-G. Methodology: IF-A, MAG-G. Formal analysis: IF-A. Data curation: MG-G, FG-B, YF-C, AG-R, AD-G, JQ-A, ID-F. Writing – original draft preparation: IF-A, MAG-G, MG-G. Writing – review & editing: IF-A, MAG-G, MG-G. Funding acquisition: IF-A. All authors contributed to the article and approved the submitted version.

## Supplementary material

Supplementary Figure 1 Frequency distribution of the three complement pathways functional assays in the SLE patients. X axes represents the percentage value of each pathway; Y axes express the number of SLE patients. Functional C pathways are shown semi-quantitatively using the optical density ratio between a positive control and the sample. While classical pathway functional test was normally distributed, the alternative and lectin routes were skewed to the left toward lower values showing, therefore, activation of these paths.

Supplementary Figure 2 Scatterplots of the correlation between C functional assays of the 3 pathways and C3 and C3a.

Supplementary Figure 3 Chord diagrams of the relationship of all C pathway functional assays and the serum molecules between them (left), and of the three C pathway functional tests to serum C elements (right). This diagram shows a many-to-many relationship between C elements and routes as curved arcs within a circle. Thickness of the arc is proportional to the significance of the flow. As it can be seen, flows or connections between nodes did not show a specific pattern from which a certain association can be inferred. CL, classical; AL, alternative; LE, lectin; fD, factor D; fH, factor H.

Supplementary Figure 4 Relation of SDI score (continuous variable) and C functional assays of the three routes. CL, classical; AL, alternative; LE, lectin.

Supplementary Figure 5 Heatmap of complete SDI items relationship to activation of C pathways and serum molecules. Values in the cells represent Spearman’s rho coefficient (* denotes p value < 0.05). Positive and negative correlations are shown in blue and red, respectively. The number of patients who met each SDI item is shown in the left margin. CL, classical; AL, alternative; LE, lectin; fD, factor D; fH, factor H. CL, LE and AL in circles refer to the functional tests of these pathways.

Supplementary Figure 6 Heatmap of complete autoantibodies and lupus anticoagulant relation to C pathways functional assays and serum molecules. Values in the cells represent Spearman’s rho coefficient (* denotes p value < 0.05). Positive and negative correlations are shown, respectively, in blue and red. The number of patients who presented each autoantibody is shown in the right margin. CL, classical; AL, alternative; LE, lectin; fD, factor D; fH, factor H.ACA: anticardiolipin antibodies, anti-beta2: anti-beta2glycoprotein antibodies; lupus antic., lupus anticoagulant. CL, LE and AL in circles refer to the functional tests of these cascades.

Supplementary Table 1 Spearman’s rho correlations between C pathways and products. In columns CL: classical pathway functional assay, LE: lectin path functional assay, AL: alternative pathway functional assay. Units are only shown in rows. Spearman’s Rho correlation index and p value are shown in cells. Significant p values are depicted in bold.

## References

[1] Holers VM. Complement and its receptors: new insights into human disease. Annu Rev Immunol (2014) 32:433–59. doi: 10.1146/annurev-immunol-032713-120154 24499275

[2] Macedo ACL Isaac L. Systemic lupus erythematosus and deficiencies of early components of the complement classical pathway. Front Immunol (2016) 7:55. doi: 10.3389/fimmu.2016.00055 26941740 PMC4764694

[3] Kao AH Navratil JS Ruffing MJ Liu CC Hawkins D McKinnon KM. Erythrocyte C3d and C4d for monitoring disease activity in systemic lupus erythematosus. Arthritis Rheum (2010) 62:837–44. doi: 10.1002/art.27267 PMC2917974 20187154

[4] Weinstein A Alexander RV. Zack DJ. A review of complement activation in SLE. Curr Rheumatol Rep (2021) 23(3):16. doi: 10.1007/s11926-021-00984-1 33569681 PMC7875837

[5] Hochberg MC. Updating the American college of rheumatology revised criteria for the classification of systemic lupus erythematosus. Arthritis Rheumatol (1997) 40:1725. doi: 10.1002/art.1780400928 9324032

[6] Gladman DD Ibañez D Urowltz MB. Systemic lupus erythematosus disease activity index 2000. J Rheumatol (2002) 29:288–91. 11838846

[7] Gladman D Ginzler E Goldsmith C Fortin P Liang M Urowitz M. The development and initial validation of the systemic lupus international collaborating Clinics/American college of rheumatology damage index for systemic lupus erythematosus. Arthritis Rheum (1996) 39:363–9. doi: 10.1002/art.1780390303 8607884

[8] Mosca M Bombardieri S. Assessing remission in systemic lupus erythematosus. Clin Exp Rheumatol 24:S-99–104. 17083771

[9] Katz JD Senegal J-L Rivest C Goulet J-R Rothfield N. A simple severity of disease index for systemic lupus erythematosus. Lupus (1993) 2:119–23. doi: 10.1177/096120339300200210 8330033

[10] Troldborg A Thiel S Trendelenburg M Friebus-Kardash J Nehring J Steffensen R. The lectin pathway of complement activation in patients with systemic lupus erythematosus. J Rheumatol (2018) 45:1136–44. doi: 10.3899/jrheum.171033 29907670

[11] Palarasah Y Nielsen C Sprogøe U Christensen ML Lillevang S Madsen HO. Novel assays to assess the functional capacity of the classical, the alternative and the lectin pathways of the complement system. Clin Exp Immunol (2011) 164:388–95. doi: 10.1111/j.1365-2249.2011.04322.x PMC3087935 21401574

[12] Arbore G Kemper C Kolev M. Intracellular complement - the complosome - in immune cell regulation. Mol Immunol (2017) 89:2–9. doi: 10.1016/j.molimm.2017.05.012 28601357 PMC7112704

[13] K.Liszewski M Atkinson JP. Complement regulators in human disease: lessons from modern genetics. J Intern Med (2015) 277:294–305. doi: 10.1111/joim.12338 25495259

[14] Vaught AJ Braunstein EM Jasem J Yuan X Makhlin I Eloundou S. Germline mutations in the alternative pathway of complement predispose to HELLP syndrome. JCI Insight (2018) 3(6):e99128. doi: 10.1172/jci.insight.99128 29563339 PMC5926944

[15] Ohmura K. Which is the best SLE activity index for clinical trials? Mod Rheumatol (2021) 31(1):20–8. doi: 10.1080/14397595.2020.1775928 32475188

[16] Ward PA. The dark side of C5a in sepsis. Nat Rev Immunol (2004) 4(2):133–42. doi: 10.1038/nri1269 15040586

[17] Lee HM Wysoczynski M Liu R Shin DM Kucia M Botto M. Mobilization studies in complement-deficient mice reveal that optimal AMD3100 mobilization of hematopoietic stem cells depends on complement cascade activation by AMD3100-stimulated granulocytes. Leukemia (2010) 24:573–82. doi: 10.1038/leu.2009.271 PMC2838235 20033053

[18] Mastellos DC DeAngelis RA Lambris JD. Complement-triggered pathways orchestrate regenerative responses throughout phylogenesis. Semin Immunol (2013) 25:29–38. doi: 10.1016/j.smim.2013.04.002 23684626 PMC3920450

[19] Merle NS Church SE Fremeaux-Bacchi V Roumenina LT. Complement system part I - molecular mechanisms of activation and regulation. Front Immunol (2015) 6. doi: 10.3389/fimmu.2015.00262 PMC4451739 26082779

[20] Markiewski MM Daugherity E Karbowniczek M Reese B. The role of complement in angiogenesis. antibodies. Antibodies (Basel) (2020) 9(4):67. doi: 10.3390/antib9040067 33271774 PMC7709120

[21] Cai YH Deng J Chen ZL Mei H Tang L Luo SS. Brief report on the relation between complement C3a and anti dsDNA antibody in systemic lupus erythematosus. Sci Rep (2022) 12(1):7098. doi: 10.1038/s41598-022-10936-z 35501405 PMC9061720

[22] Iwasaki T Doi H Tsuji H Tabuchi Y Hashimoto M Kitagori K. Phenotypic landscape of systemic lupus erythematosus: An analysis of the Kyoto lupus cohort. Mod Rheumatol (2022) 32:571–6. doi: 10.1093/mr/roab020 34894258

[23] Hubbard EL Pisetsky DS Lipsky PE. Anti-RNP antibodies are associated with the interferon gene signature but not decreased complement levels in SLE. Ann Rheum Dis 81:632–43. doi: 10.1136/annrheumdis-2021-221662 35115332

[24] Foley JH Conway EM. Cross talk pathways between coagulation and inflammation. Circ Res (2016) 118(9):1392–408. doi: 10.1161/CIRCRESAHA.116.306853 27126649

[25] Breen KA Seed P Parmar K Moore GW Stuart-Smith SE Hunt BJ. Complement activation in patients with isolated antiphospholipid antibodies or primary antiphospholipid syndrome. Thromb Haemost (2012) 107:423–9. doi: 10.1160/TH11-08-0554 22234447

[26] Oku K Atsumi T Bohgaki M Amengual O Kataoka H Horita T. Complement activation in patients with primary antiphospholipid syndrome. Ann Rheum Dis (2009) 68:1030–5. doi: 10.1136/ard.2008.090670 18625630

[27] Yin D de Laat B Devreese KMJ Kelchtermans H. The clinical value of assays detecting antibodies against domain I of β2-glycoprotein I in the antiphospholipid syndrome. Autoimmun Rev (2018) 17:1210–8. doi: 10.1016/j.autrev.2018.06.011 30316989

[28] Kojima T Inoue D Wajima T Uchida T Yamada M Ohsawa I. Circulating immune-complexes and complement activation through the classical pathway in myeloperoxidase-ANCA-associated glomerulonephritis. Ren Fail (2022) 44(1):714–23. doi: 10.1080/0886022X20222068445 PMC9067964 35491890

[29] Selvaskandan H Kay Cheung C Dormer J. Inhibition of the lectin pathway of the complement system as a novel approach in the management of IgA vasculitis-associated nephritis. Nephron (2020) 144:453–8. doi: 10.1159/000508841. 32721954

[30] Khan AH Pierce CO De Salvo G Griffiths H Nelson M Cree AJ. The effect of systemic levels of TNF-alpha and complement pathway activity on outcomes of VEGF inhibition in neovascular AMD. Eye (2021) 36:11. doi: 10.1038/s41433-021-01824-3 PMC9581945 34750590

[31] Holter JC Pischke SE de Boer E Lind A Jenum S Holten AR. Systemic complement activation is associated with respiratory failure in COVID-19 hospitalized patients. Proc Natl Acad Sci U.S.A. (2020) 117:25018–25. doi: 10.1073/pnas.2010540117 PMC7547220 32943538

[32] Diatlov D Bohorquez A Jackson M Cheong M Kahr WHA Kuo KHM. Pediatric sickle cell disease: A potential role for the complement system. Blood (2022) 140:2517–8. doi: 10.1182/blood-2022-167696

