# Supplementary figures and images for: Full characterization of the three pathways of the complement system in patients with systemic lupus erythematosus
Source: Front Immunol. 2023 Apr 21;14:1167055. doi: 10.3389/fimmu.2023.1167055 (PMC10160460; doi:10.3389/fimmu.2023.1167055)

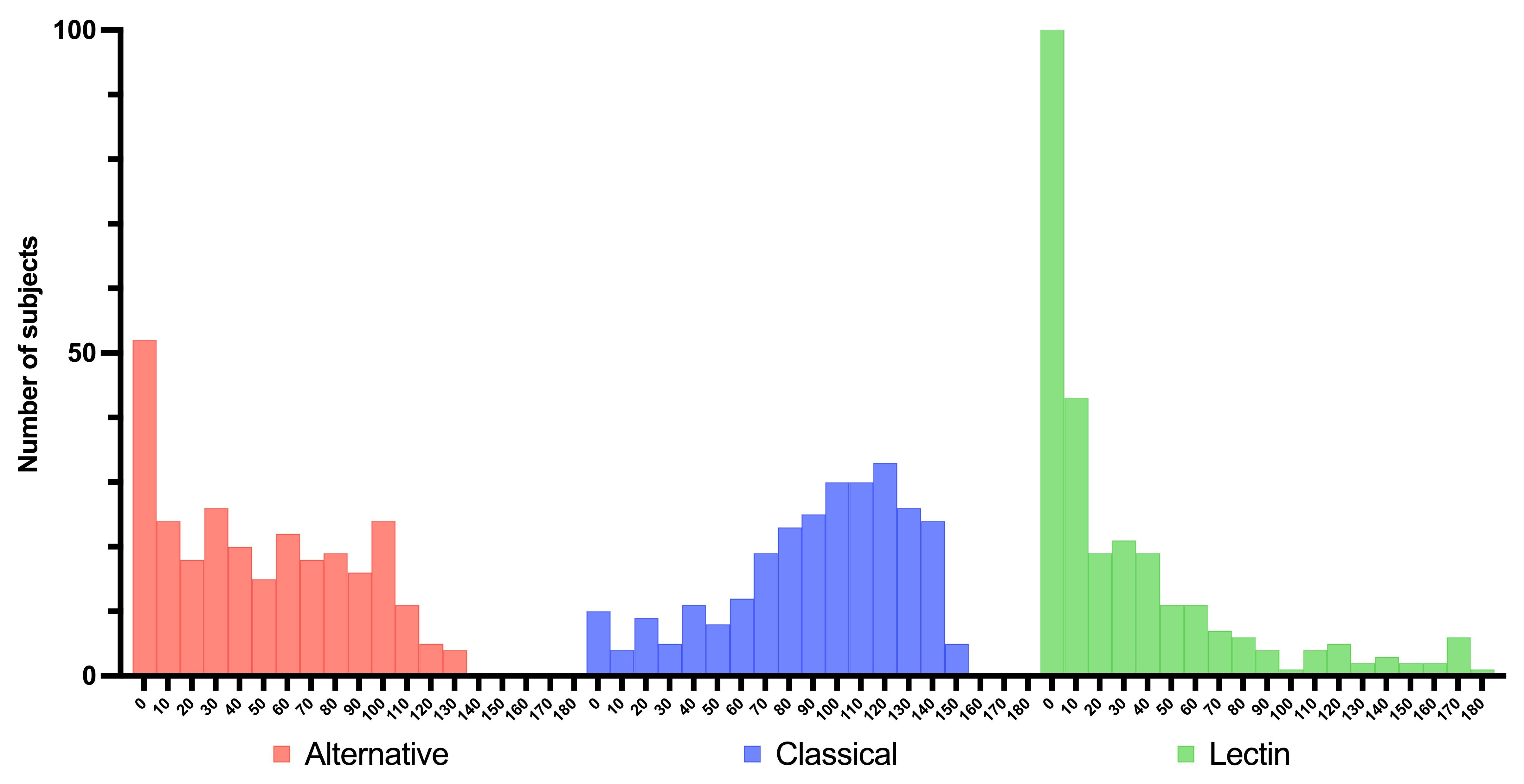

Supplement: Supplementary Figure 1 — Frequency distribution of the three complement pathways functional assays in the SLE patients. X axes represents the percentage value of each pathway; Y axes express the number of SLE patients. Functional C pathways are shown semi-quantitatively using the optical density ratio between a positive control and the sample. While classical pathway functional test was normally distributed, the alternative and lectin routes were skewed to the left toward lower values showing, therefore, activation of these paths. [file Image_1.jpeg]

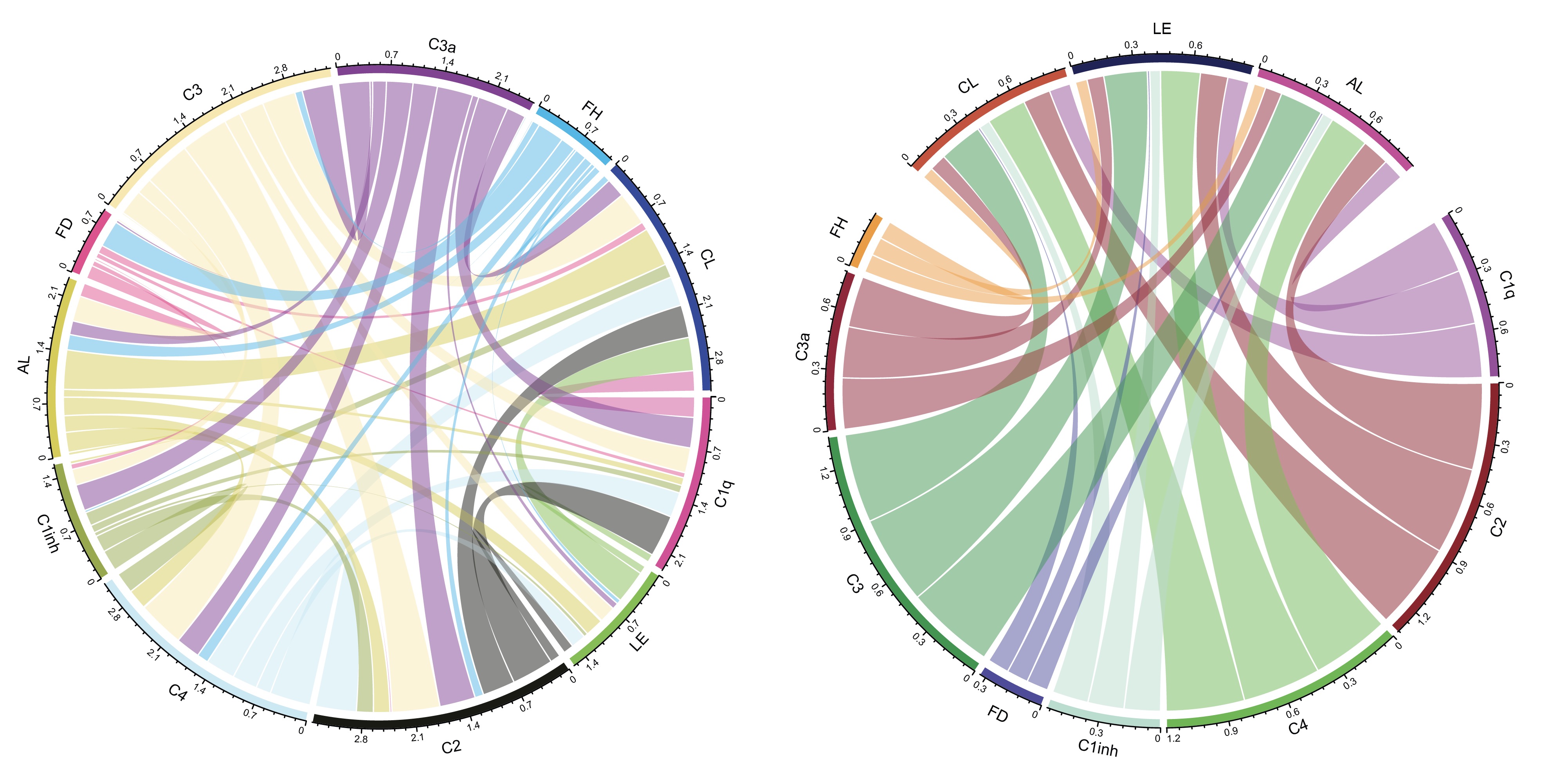

Supplement: Supplementary Figure 3 — Chord diagrams of the relationship of all C pathway functional assays and the serum molecules between them (left), and of the three C pathway functional tests to serum C elements (right). This diagram shows a many-to-many relationship between C elements and routes as curved arcs within a circle. Thickness of the arc is proportional to the significance of the flow. As it can be seen, flows or connections between nodes did not show a specific pattern from which a certain association can be inferred. CL, classical; AL, alternative; LE, lectin; fD, factor D; fH, factor H. [file Image_3.jpeg]

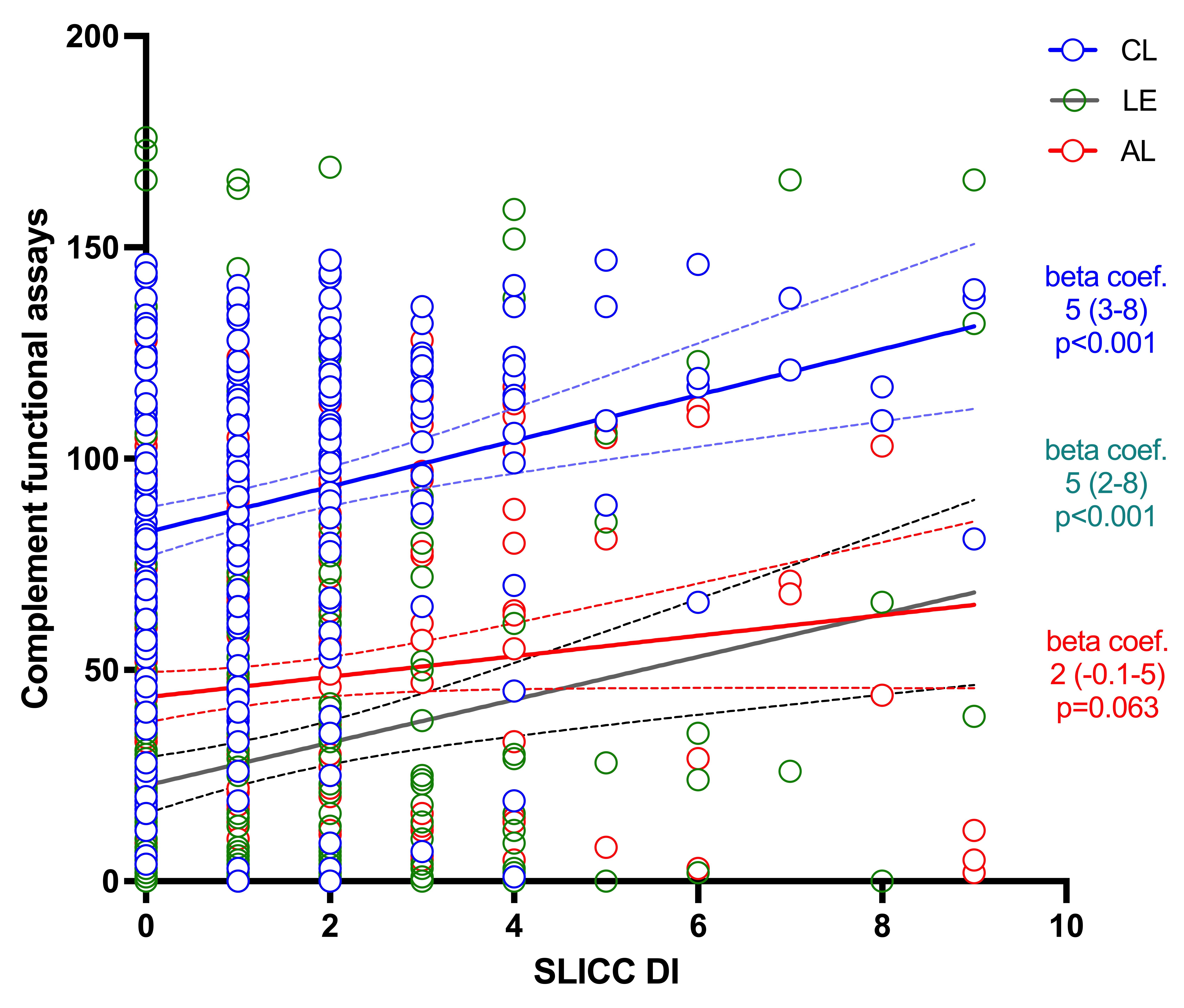

Supplement: Supplementary Figure 4 — Relation of SDI score (continuous variable) and C functional assays of the three routes. CL, classical; AL, alternative; LE, lectin. [file Image_4.jpeg]

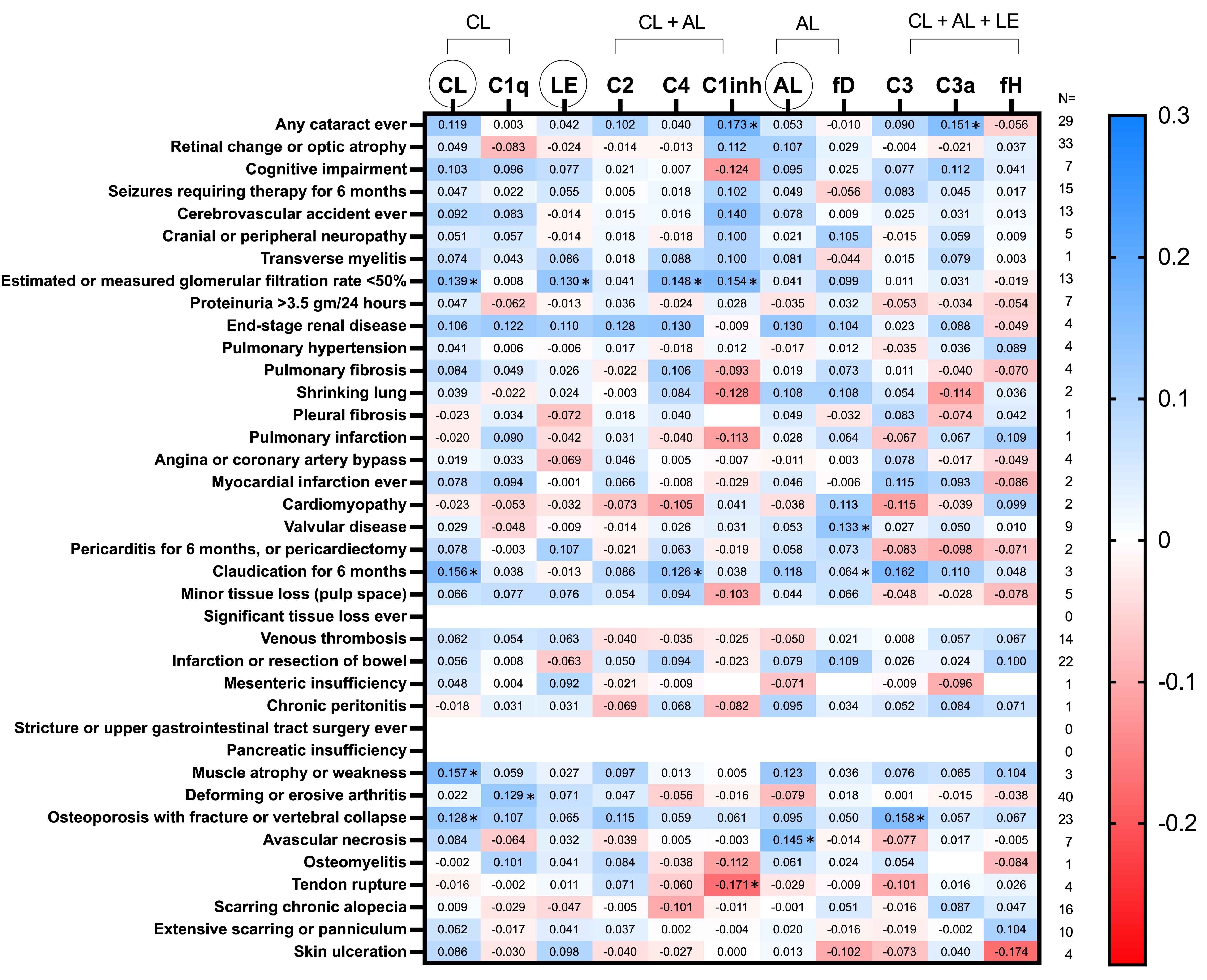

Supplement: Supplementary Figure 5 — Heatmap of complete SDI items relationship to activation of C pathways and serum molecules. Values in the cells represent Spearman’s rho coefficient (* denotes p value < 0.05). Positive and negative correlations are shown in blue and red, respectively. The number of patients who met each SDI item is shown in the left margin. CL, classical; AL, alternative; LE, lectin; fD, factor D; fH, factor H. CL, LE and AL in circles refer to the functional tests of these pathways. [file Image_5.jpeg]

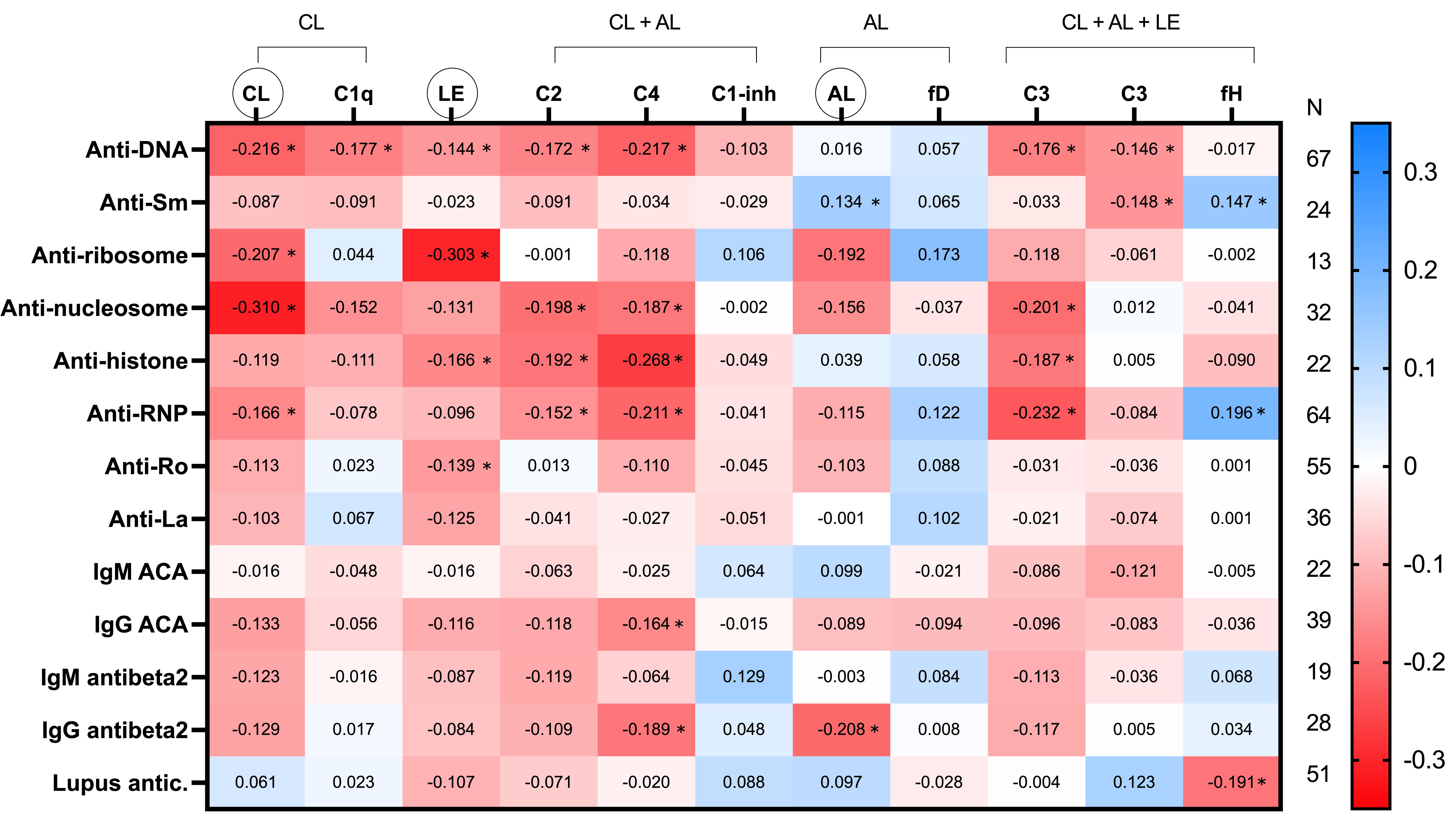

Supplement: Supplementary Figure 6 — Heatmap of complete autoantibodies and lupus anticoagulant relation to C pathways functional assays and serum molecules. Values in the cells represent Spearman’s rho coefficient (* denotes p value < 0.05). Positive and negative correlations are shown, respectively, in blue and red. The number of patients who presented each autoantibody is shown in the right margin. CL, classical; AL, alternative; LE, lectin; fD, factor D; fH, factor H.ACA: anticardiolipin antibodies, anti-beta2: anti-beta2glycoprotein antibodies; lupus antic., lupus anticoagulant. CL, LE and AL in circles refer to the functional tests of these cascades. [file Image_6.jpeg]
