# Supplementary material for: Full characterization of the three pathways of the complement system in patients with systemic lupus erythematosus
Source: Front Immunol. 2023 Apr 21;14:1167055. doi: 10.3389/fimmu.2023.1167055 (PMC10160460; doi:10.3389/fimmu.2023.1167055)
Supplement: Supplementary Table 1 [file Table_1.docx]

| **Supplementary Table 1. Spearman's rho correlations between C pathways and products.** | | | | | | | | |  | |  | |  | |  | |  |  |
| --- | --- | --- | --- | --- | --- | --- | --- | --- | --- | --- | --- | --- | --- | --- | --- | --- | --- | --- |
|  |  | Classical path | | Lectin path | Common of the CL and LE paths | | | Alternative path | | | | Common paths | | | | | |  |
|  |  | CL | C1q | LE | C2 | C4 | C1-inh | AL | | Factor D | | C3 | | C3a | | Factor H | |  |
| Classical path | |  |  |  |  |  |  |  | |  | |  | |  | |  | |  |
|  | Functional assay | - |  |  |  |  |  |  | |  | |  | |  | |  | |  |
|  | C1q, mg/dl | **0.274** |  |  |  |  |  |  | |  | |  | |  | |  | |  |
|  |  | **<0.001** |  |  |  |  |  |  | |  | |  | |  | |  | |  |
| Lectin path | |  |  |  |  |  |  |  | |  | |  | |  | |  | |  |
|  | Functional assay | **0.511** | **0.178** |  |  |  |  |  | |  | |  | |  | |  | |  |
|  |  | **<0.001** | **0.019** |  |  |  |  |  | |  | |  | |  | |  | |  |
| Classical and lectin paths | |  |  |  |  |  |  |  | |  | |  | |  | |  | |  |
|  | C2, mg/dl | **0.408** | **0.602** | **0.177** |  |  |  |  | |  | |  | |  | |  | |  |
|  |  | **<0.001** | **<0.001** | **0.020** |  |  |  |  | |  | |  | |  | |  | |  |
|  | C4, mg/dl | **0.397** | **0.443** | **0.333** | **0.614** |  |  |  | |  | |  | |  | |  | |  |
|  |  | **<0.001** | **<0.001** | **<0.001** | **<0.001** |  |  |  | |  | |  | |  | |  | |  |
|  | C1 inhibitor, mg/dl | **0.186** | 0.105 | 0.080 | **0.223** | **0.294** |  |  | |  | |  | |  | |  | |  |
|  |  | **0.015** | 0.170 | 0.295 | **0.003** | **<0.001** |  |  | |  | |  | |  | |  | |  |
| Alternative path | |  |  |  |  |  |  |  | |  | |  | |  | |  | |  |
|  | Functional assay | **0.523** | 0.102 | **0.338** | **0.174** | **0.303** | 0.000 |  | |  | |  | |  | |  | |  |
|  |  | **<0.001** | 0.183 | **<0.001** | **0.023** | **<0.001** | 0.997 |  | |  | |  | |  | |  | |  |
|  | Factor D, ng/ml | 0.115 | -0.023 | -0.005 | 0.025 | -0.001 | 0.075 | 0.121 | |  | |  | |  | |  | |  |
|  |  | 0.133 | 0.764 | 0.953 | 0.748 | 0.986 | 0.328 | 0.114 | |  | |  | |  | |  | |  |
| Common paths | |  |  |  |  |  |  |  | |  | |  | |  | |  | |  |
|  | C3, mg/dl | **0.433** | **0.424** | **0.286** | **0.708** | **0.599** | **0.241** | **0.286** | | **0.023** | |  | |  | |  | |  |
|  |  | **<0.001** | **<0.001** | **<0.001** | **<0.001** | **<0.001** | **0.002** | **<0.001** | | **0.770** | |  | |  | |  | |  |
|  | C3a, mg/dl | **0.238** | **0.440** | **0.196** | **0.465** | **0.333** | **0.365** | **0.181** | | 0.001 | | **0.481** | |  | |  | |  |
|  |  | **0.002** | **<0.001** | **0.010** | **<0.001** | **<0.001** | **<0.001** | **0.017** | | 0.987 | | **<0.001** | |  | |  | |  |
|  | Factor H, ng/ml x10e-3 | 0.135 | 0.102 | 0.086 | **0.226** | 0.147 | -0.009 | **0.202** | | **0.328** | | 0.109 | | 0.070 | |  | |  |
|  |  | 0.078 | 0.181 | 0.262 | **0.003** | 0.054 | 0.911 | **0.0079** | | **<0.001** | | 0.155 | | 0.359 | | **-** | |  |

In columns CL: classical pathway functional assay, LE: lectin path functional assay, AL: alternative pathway functional assay. Units are only shown in rows. Spearman’s Rho correlation index and p value are shown in cells. Significant p values are depicted in bold.
